# Telemedicine in Brazil: Teleconsultations at the Largest University Hospital in the Country

**DOI:** 10.1089/tmr.2023.0012

**Published:** 2023-07-31

**Authors:** Paula Gobi Scudeller, Antônio José Pereira, Giovanni Guido Cerri, Fábio Biscegli Jatene, Marco Bego, Talita Freitas Amaral, Michelle Louvaes Garcia, Celina Almeida Lamas, Aline Morgan Alvarenga, Marco Antônio Gutierrez, Vilson Cobello Junior, Carlos Roberto Ribeiro de Carvalho

**Affiliations:** ^1^ Digital Health, Technological Innovation Hub (InovaHC), Hospital das Clínicas, Faculdade de Medicina, Universidade de São Paulo (HCFMUSP), São Paulo, Brazil.; ^2^ Pulmonary Division, Heart Institute (InCor), Hospital das Clínicas, Faculdade de Medicina, Universidade de São Paulo (HCFMUSP), São Paulo, Brazil.; ^3^ Hospital das Clínicas, Faculdade de Medicina, Universidade de São Paulo (HCFMUSP), São Paulo, Brazil.; ^4^ Radiology Institute (InRad), Hospital das Clínicas, Faculdade de Medicina, Universidade de São Paulo (HCFMUSP), São Paulo, Brazil.; ^5^ Heart Institute (InCor), Hospital das Clínicas, Faculdade de Medicina, Universidade de São Paulo (HCFMUSP), São Paulo, Brazil.; ^6^ Informatics Division, Heart Institute (InCor), Hospital das Clínicas, Faculdade de Medicina, Universidade de São Paulo (HCFMUSP), São Paulo, Brazil.; ^7^ Núcleo Especializado em Inovação Tecnológica, Hospital das Clínicas, Faculdade de Medicina, Universidade de São Paulo (HCFMUSP), São Paulo, Brazil.

**Keywords:** digital health, telemedicine, teleconsultation service, telehealth

## Abstract

The coronavirus disease (COVID-19) pandemic leveraged telemedicine worldwide mainly due to the need for social distancing, patient safety, and infection prevention. The Hospital das Clínicas da Faculdade de Medicina da Universidade de São Paulo (HCFMUSP) was a key reference site in the treatment of COVID-19 severe cases in the country. To continue patient's health care, it became necessary to increase the number of teleconsultations and standardize it institutionally. Herein, we briefly described how the HCFMUSP improved the teleconsultation health care service during the COVID-19 pandemic, highlighting the implementation of important innovations and the throughout standardization process, including patients and professional workflow. We also detailed the methodology used to implement or improve teleconsultation in a medical/multidisciplinary specialty at HCFMUSP. All these efforts made the HCFMUSP reach the goal of converting 15% of all face-to-face consultations into teleconsultations only in 2021. In addition, there were more than 370,000 teleconsultations until the end of 2022. Our experience has shown that having a supporting team, a digital certification process, and the data integration were key factors toward the successful implementation of the teleconsultation services. We believe that progressing toward teleconsultation will improve the population covered by health care services in Brazil, as well as contribute to a reduction of waiting time, and solving costs to health care institutions and patients. We expect this report of our experience in teleconsultation implementation could inspire and guide other health care institutions in the development of telemedicine.

## Introduction

In March 2020, the World Health Organization declared a pandemic caused by the coronavirus disease (COVID-19), and the Hospital das Clínicas da Faculdade de Medicina da Universidade de São Paulo (HCFMUSP) was a key reference site in the treatment of COVID-19 severe cases in Brazil.^1^ Even before the pandemic, it was considered one of the most important Brazilian health care centers for producing techno-scientific information and promote their dissemination, representing the best public hospital in Brazil, with excellence in teaching, research, and health care assistance.^2,3^

The HCFMUSP is part of the Brazilian Unified Health System (Sistema Único de Saúde—SUS). The SUS was created in 1988 and is responsible for providing free access to health care services to all Brazilian citizens. It is one of the largest public health systems in the world, covering more than 200 million people. Almost 71.5% of Brazilians do not have any private health insurance, and they rely on SUS for medical treatments, health assistance, and other health care services.^4^

In addition, Brazil is a large and diverse country, with vast differences in geography, economic development, and population density, which affect access to health care.^5^ In this context, telehealth projects emerged, with the purpose of facilitating access to health services, by expanding and speeding up diagnosis and early detection of diseases.^6–8^

HCFMUSP provides medical care locally, in the state of São Paulo, but also to people coming from all over the country. For example, it was carried out around 1.3 million outpatient medical consultations in different specialties; 125 thousand emergency services; 11.8 million pathological exams; and 842.4 thousand imaging exams only in 2019.^9^ During the pandemic, teleconsultation favored social distancing, ensuring the safety of all patients involved.^8^ It also contributed to a significant reduction in the flux of people visiting the HCFMUSP hospital complex.^10^

However, at that time, there was no approved regulation for the practice of teleconsultation in Brazil. While facing a public health emergency of huge proportions, the Brazilian Ministry of Health, in a joint effort, formulated and approved guidelines for the telehealth practice.^11^ Thus, in December of 2022, the Brazilian Federal Government published the law N°14.510, which authorizes and regulates telehealth practices in the country.^12^ Therefore, we aimed herein at detailing the implementation of teleconsultations in the HCFMUSP, presenting goals and perspectives so far.

## Teleconsultations Leveraged During the COVID-19 Pandemic at HCFMUSP

The HCFMUSP is composed of eight institutes divided according to the assistance area, including the Cancer Institute (*Instituto do Câncer do Estado de São Paulo—ICESP*), the Central Institute (*Instituto Central do Hospital das Clínicas—ICHC*), the Children Institute (*Instituto da Criança—ICR*), Heart Institute (*Instituto do Coração—InCor*), the Orthopedic Institute (*Instituto de Ortopedia—IOT*), the Physical Medicine and Rehabilitation Institute (*Instituto de Medicina Física e Reabilitação—IMREA*), and the Psychiatry Institute (*Instituto de Psiquiatria—IPq*) and Radiology Institute (*Instituto de Radiologia—InRad*).

Teleconsultations were already practiced in 7 institutes of the HCFMUSP, even before the pandemic. At that time, teleconsultation was not a standardized institutional program, which complicated the self-evaluation and the monitoring process. During the pandemic, it became necessary to increase the number of teleconsultations due to the need for social distancing. Therefore, a Digital Health Board was created as an initial step toward the institutional implementation of teleconsultation in the HCFMUSP complex.

This group included the following members, the Chief Executive Officer of HCFMUSP and leaders of 9 institutes, involving the medical and the administrative directive committee of HCFMUSP, which was essential for the acceptance of teleconsultation practice, mainly because the general animosity regarding remote consultations required a cultural change in the institution.

In the beginning of 2020, a partnership between the HCFMUSP and the Global Better Health Programme (BHP-UK) was established. In detail, the “Global Better Health programme—BHP”^13^ aims at addressing the growing burden of Non-Communicable Diseases worldwide. It also aims at strengthening local health system structures by investing £79.3 million into 5 key areas and providing technical assistance to support their objectives.

Among the strategic endorsed areas, there are digital health initiatives, education, and training of health care professionals. HCFMUSP was already projecting and implementing digital health initiatives in the Brazilian public health system at that time. Therefore, HCFMUSP was selected by the BHP program to participate in a consultancy process by a third-party company to map those initiatives and help the hospital complex to implement and mature the ongoing digital health projects. In this partnership, 20 digital health solutions were proposed (Table 1), aiming at the increase of efficiency and the overall quality in patient health care at HCFMUSP, and then expand throughout the SUS network. It is important to emphasize that teleconsultation was the first initiative highlighted by the program.

**Table 1.** Listed Digital Health Solutions to Be Implemented at the Hospital das Clínicas da Faculdade de Medicina da Universidade de São Paulo

HCFMUSP focus	Core	Solutions	
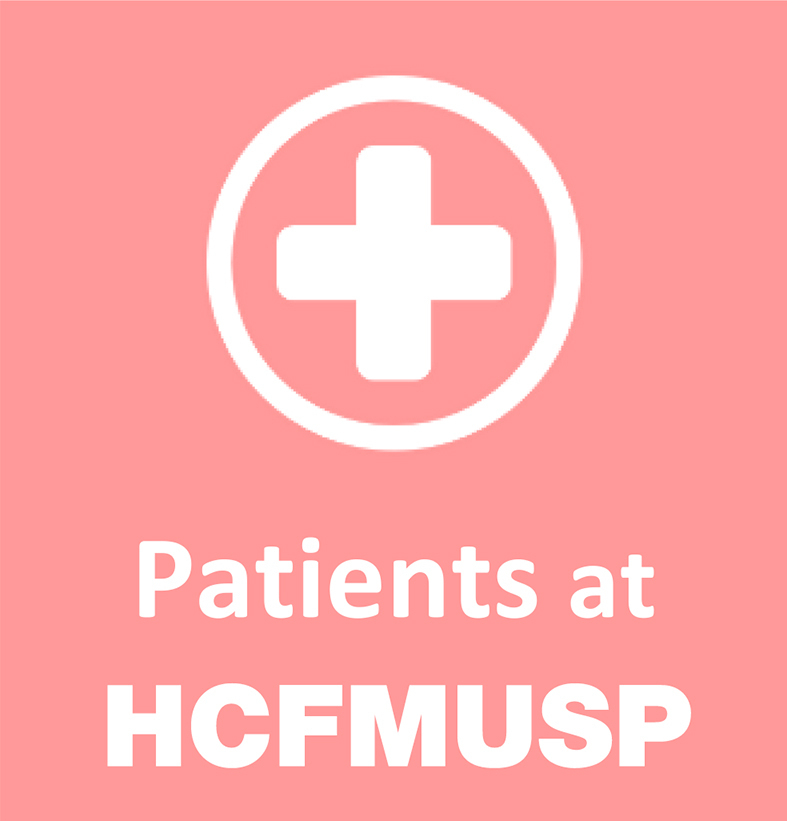	1 Improve patients experience at the HCFMUSP	• Provide teleconsultation for a range of medical specialties	• Implement interoperability and expand the functionalities of the patient's electronic medical records
		• Translate patient assistance in a digital health service	• Use of virtual reality for the long-term health care support
		• Remote patient's visit	• Create a bureau specialized in improvement of digital patient's journey
		• Implement telerehab	
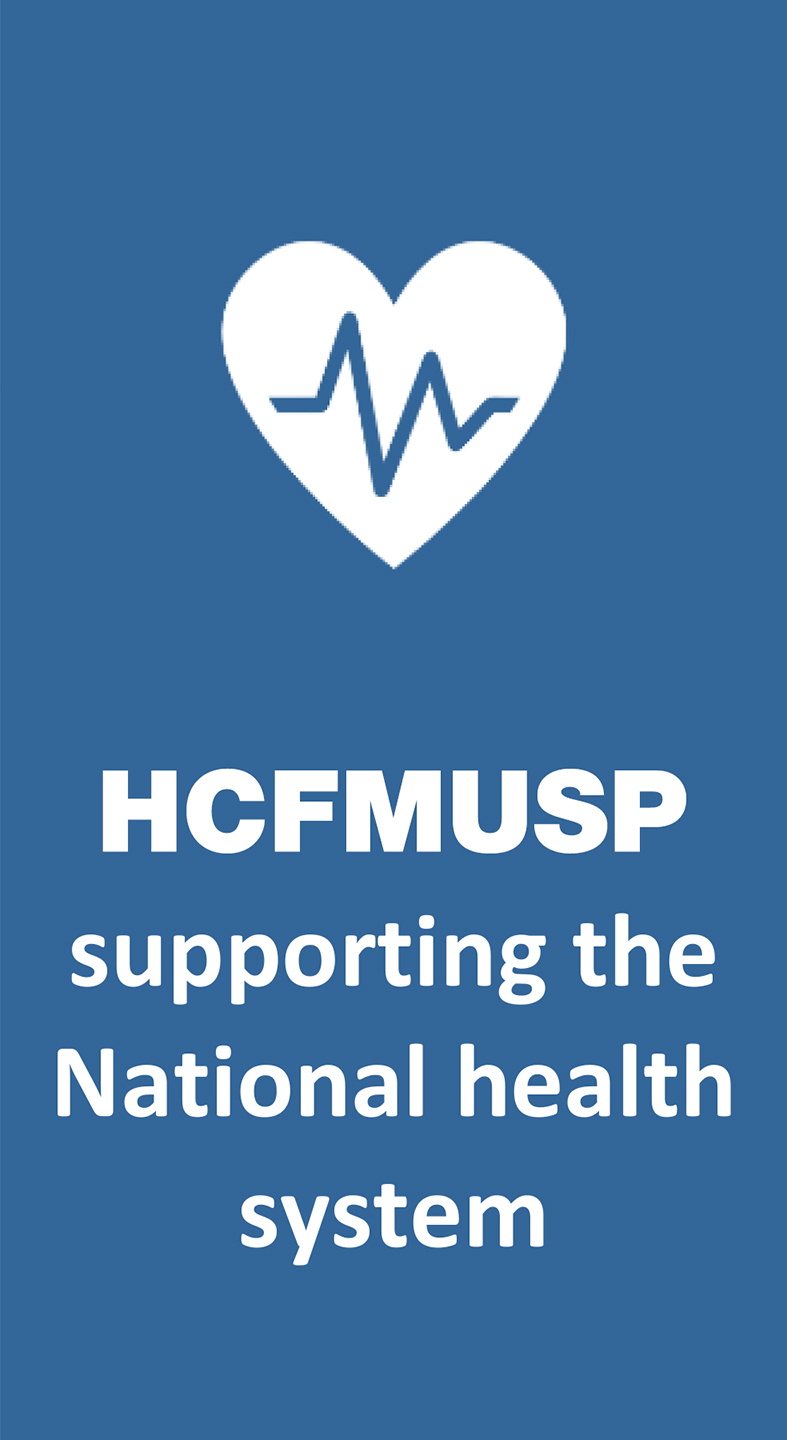	2 Increase the capacity of the public national health system	• Integrate a health care team	• Implement telemonitoring and remote access to imaging health tech.
		• Provide several medical specialties in teleconsulting services	• Provide teleintervention in emergency cases
	3 Implement innovative solutions in digital health	• Telemonitoring of patients with chronic diseases	• Teletraining in surgery centers (including use of virtual reality)
		• Use of AI for rapid imaging exams diagnosis	• Run pilot projects in primary care
		• Use of AI for tracking the patient's journey and streamline future assistance	• Run pilot projects in Telemonitoring for patients with chronic diseases
		• Promote continuous education by developing training for ICU	• Create a portfolio of digital health services and implement in the public health system
			• Create a continuous education process in telemedicine

AI, artificial intelligence; HCFMUSP, Hospital das Clínicas da Faculdade de Medicina da Universidade de São Paulo; ICU, intensive care unity.

The first step included carrying out a proof of concept for teleconsultations. For this, the board of directors of the HCFMUSP, together with the Digital Health Board verified the capacity to perform teleconsultations, mapped the eligible specialties, as well as the current structure and the equipment needed, and then chose a “Focal Point” from each medical specialty responsible for implementing the initiative.

Basically, “Focal Point” is defined as an institutional leader of a medical specialty who is responsible to report the state of the digital health implementation process in his field at the designated institute of HCFMUSP. The teleconsultations occurred preferentially by videoconferences. As a management strategy, weekly meetings were scheduled with the focal point to monitor the status report of the implementation of the teleconsultations and to adjust it according to each medical specialty.

## Innovation Tools and Process Standardization for Teleconsultations at HCFMUSP

The standardization of the teleconsultation process involved the development of an institutional platform for teleconferences (iConf).^14^ This decision was driven by concerns related to controlling the security and privacy of patient data within the HCFMUSP, but also ponder over the costs involved in the acquisition of a commercially available platform. By using a platform developed *in house*, the institution could ensure that all data registered in the services were stored securely and not shared or manipulated by third parties. This is extremely important in health care, where sensitive personal data, test results, and patient diagnoses must be kept confidential.

iConf offers a range of features that are useful for teleconsultations, including audio and video sharing, allows sharing presentations with extended whiteboard functionalities, such as pointer, zoom, and drawing tools, as well as public and private chat, screen sharing, and voice channels over the network with Internet Voice Over Internet Protocol (VoIP). The platform supports the presentation of documents in PDF and Microsoft Office formats, which is also a helpful feature for clinicians to share information with patients during a teleconsultation.

iConf platform was developed to provide real-time communication capabilities for telemedicine sessions through a standard Hypertext Mark-up Language user interface. Therefore, the platform is supported by many web browsers like Google Chrome, Firefox, Opera, and Safari. All communication between participants is encrypted using the Secure Socket Layer protocol. In addition to these features, the iConf also allows recording of telemedicine sessions, which can be useful for auditing and traceability purposes, as it allows health care providers to review past sessions and maintain records of patient care.

iConf is allocated in two web pages/mobile apps, named “Patient Portal” and the “HC at Home,” which were developed specifically to facilitate the patients' and health care professionals' access to the patient's journey, respectively. These web pages/mobile apps hosted information regarding scheduled consultations and exams, exam results, prescriptions, and an instruction guide. They also have a “teleconsultation tab,” where the patients and health care professionals can visualize the scheduled teleconsultations and access the video conference room.

In addition, for the patient to access the iConf system, they need to agree on the institutional terms of free and informed consent; otherwise, their admission to the videoconference room was blocked. After completing the teleconsultation, the patient received a satisfaction survey to answer about our service. This strategy allowed us to periodically verify the quality of the services provided through the teleconsultation aiming at its improvement.

Behind the teleconsultations, there was a multi-professional supporting team responsible for scheduling the teleconsultations, including send an appointment reminder to the patient on the day of the teleconsultation. In detail, an iConf link was sent to the patient before the teleconsultation, which allowed them to test their access to the platform and to provide individualized support in case of any technological issue arise. There was also a Technology Information (TI) department responsible for maintenance of computers and system operations, and to allow the virtual environment ready for the teleconsultation.

The HCFMUSP decided to create a “Digital Certification Process” to guarantee the teleconsultation in a secure and efficient virtual environment. For this, several measures were defined and validated, which included the use of the two institutional web portals (Patients' portal and Hospital das Clínicas-portal) for registered patients and health care professionals, individual security keys for the health care professionals involved in the teleconsultation and required for releasing electronic prescriptions, and for using electronic systems for handling medical records and for digital scheduling.

One important ongoing step is the teleconsultation data integration. For this, the HCFMUSP TI department developed a unified dashboard that assemble and compile information and indicators to be evaluated, allowing a proper visualization of the teleconsultation implementation and help monitoring the process. In detail, the data compiled of each institute in the designated dashboards allowed the visualization of the number of teleconsultations performed, the absenteeism rate, the average time of teleconsultation, the type of service employed (telephone calls or videoconference), and the results obtained from the satisfaction survey.

## Implement and Improve Teleconsultation Considering Medical Specialties at HCFMUSP

The process for implementing teleconsultation at HCFMUSP was designed by considering that digital transformation initiatives require changes in the mindset of health care professionals, as well as in the institutional values and principles.^15^ At the HCFMUSP, medical specialties verified the need to start the implementation of teleconsultation practices.

As the demand increased, the recently created Digital Health Board started to manage and organize the individual teleconsultation initiatives, to improve and standardize the practice across the whole hospital complex. Briefly, new recruited support professionals received an onboarding training in good practices in telehealth using institutional standardized guidelines created with the purpose to help them get their first steps in the teleconsultation.

In addition, to optimize the teleconsultation process, the HCFMUSP prepared a manual for patients and health care professionals, explaining in detail the teleconsultation procedures and its importance.^16^ *Hands-on* workshops were performed at the HCFMUSP to help implement teleconsultation in the institutes of pre-determined medical specialties. The content of the workshops included the introduction of good practices in telehealth, the national regulation on telehealth and the respective bill, as well as practical training activities in teleconsultation.

In regard to the content of the bill covered during the workshops, it provides guidance on the autonomy of the health care professionals involved in the telehealth activity, the formal agreement at disposal of the patient, the right for refusal for both parts and replacement for face-to-face consultations, the imbued values in health care assistance and treatment, and the confidentiality of the patient's data. In addition, HCFMUSP produced an online asynchronous training on digital health with 20 h of duration, and fully dedicated to all health care professionals requiring qualification in the topic.

Focusing on the workshops and meetings, they were designed aiming at the engagement of health care professionals in the development and in the decision-making process for a rapid teleconsultation implementation in the most demanding medical specialties of HCFMUSP. For this, a decision tree strategy helped define patient eligibility criteria and guide the overall teleconsultation implementation process.

The workshops resulted in the definition and selection of additional focal points responsible for sharing the decision tree with other professionals within the medical specialty and to adjust it in their work routine. Follow-up meetings were held to ensure the decision tree's feasibility and to collect feedback, updates, and status reports. In addition to the improvement of the teleconsultation workflow in the medical specialty, the workshops allowed to increase the health care professional's perception of the benefits in adopting digital strategies, and bringing digital health and remote assistance to patient care.

This was an important goal, as digital technologies can greatly enhance health care delivery and improve the patient outcomes. By involving health care professionals in the development and implementation of digital health strategies, the workshops increased the professional's adhesion and facilitated technological integration into clinical practice.

The conversion from face-to-face consultation into teleconsultation depends on several factors, including the particularity of each medical specialty, the patient profile, the available infrastructure, and the technological capacity. The experience acquired by the HCFMUSP professionals in digital health affairs allowed us to figure out some essential patients' eligibility criteria to perform teleconsultations. The patient must stay in a private room with a stable internet connection. If the patient is under 18 years old, elderly, or with any type of visual, hearing, or motor difficulty, the presence of a companion to assist at the time of the teleconsultation is important.

**FIG. 1.**
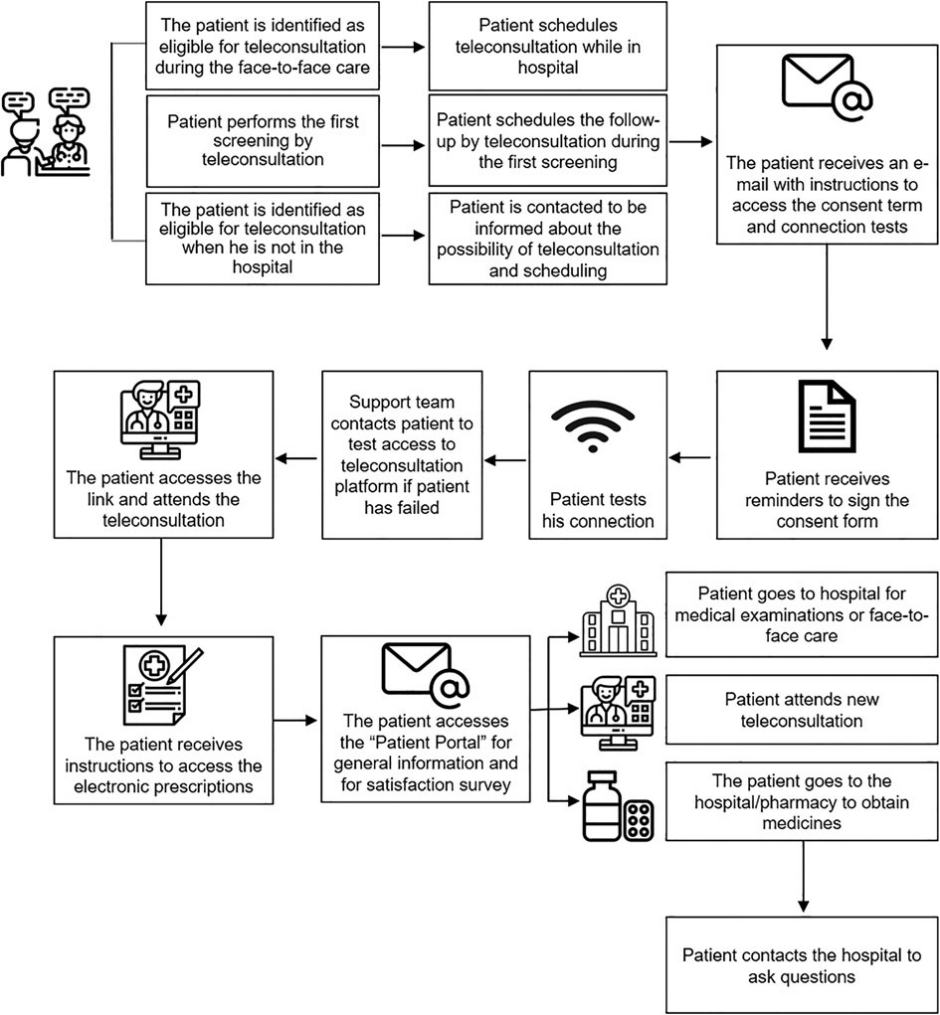
Flowchart of the journey of the patients to carry out teleconsultations at HCFMUSP. HCFMUSP, Hospital das Clínicas da Faculdade de Medicina da Universidade de São Paulo.

**FIG. 2.**
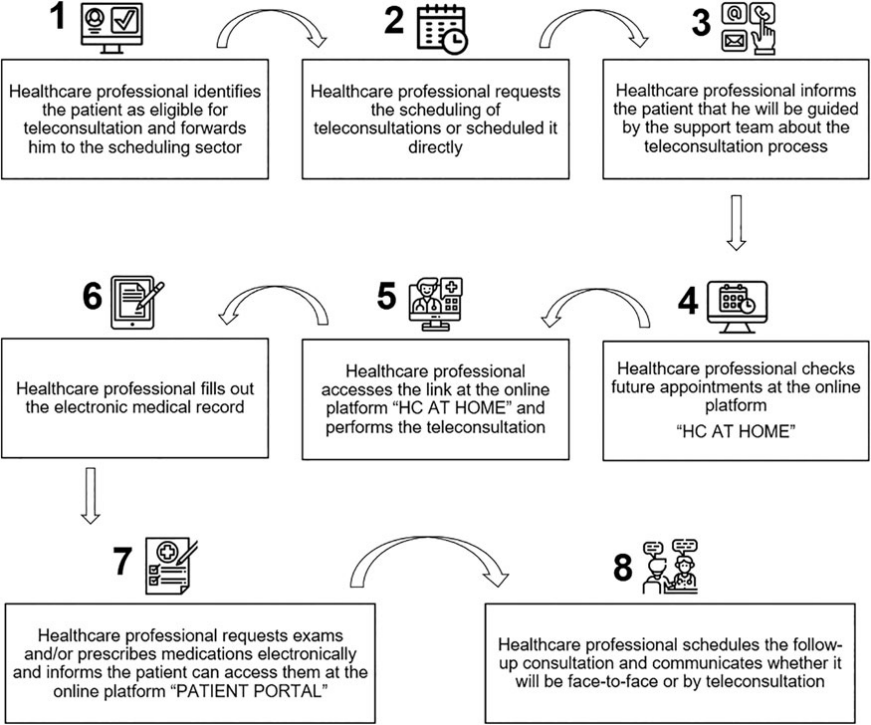
Workflow of health care professionals to carry out teleconsultations at HCFMUSP.

The journey of the patients and health care professionals involved in the teleconsultations can be seen in Figures 1 and 2.

## Teleconsultation Goals at HCFMUSP

The ambitious endeavor of HCFMUSP is implementing, optimizing, and expanding the health care assistance in the Brazilian public health system, leading the institution setting the goal of converting 15% of face-to-face consultations into teleconsultations by 2021. As a result of this institutional effort, this goal was reached in 2021, considering a sum of all teleconsultations performed in the entire institution. The multi-professional specialties (psychology, nutrition, and nursing) showed a higher conversion rate when compared with medical specialties. We verified that medical clinic was the specialty with the highest adherence to the teleconsultation practices among all available medical specialties at the HCFMUSP.

By the end of 2022, 52 workshops with a digital health and teleconsultation scope were performed, totalizing more than 100 working hours and involving more than 215 health care specialists. Also, more than 2000 health care professionals were certificated for electronic prescriptions. These efforts resulted in more than 370,000 teleconsultations, including more than 180,000 registered patients and 11,322,144 virtual interactions through the Patient Portal by the end of 2022.

**FIG. 3.**
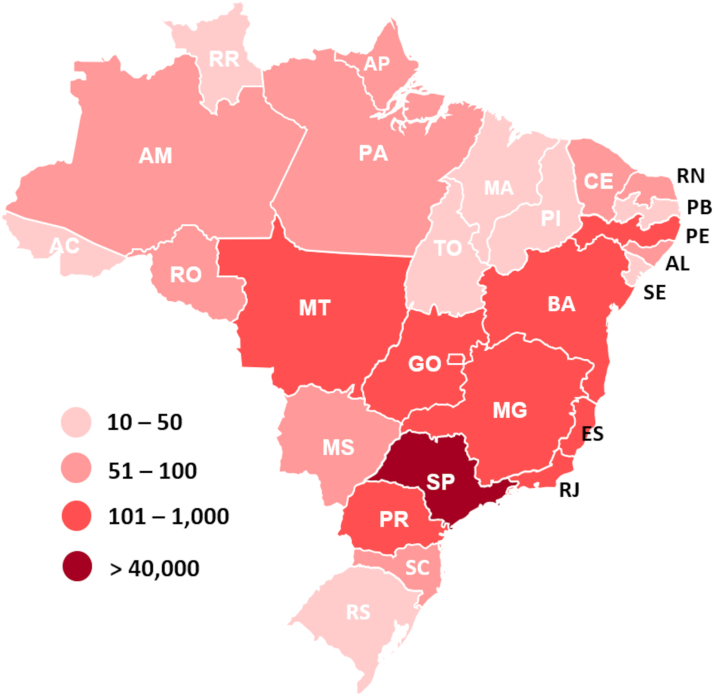
Number of teleconsultations performed by the HCFMUSP team in the year of 2022 per Brazilian state. The map was color coded to indicate the lowest value of teleconsultation performed (*slightly pink*, 10–50), to the highest number (*dark red*, >40,000).

Table 2 shows the number of teleconsultations performed at HCFMUSP in 2022 per institute, and Figure 3 demonstrates the regional distribution of these teleconsultations in the country, covering all the 26 states and the national capital. Table 3 describes the medical and multi-professional specialties that performed teleconsultation by the end of 2022.

**Table 2.** Teleconsultation in Numbers at Hospital das Clínicas da Faculdade de Medicina da Universidade de São Paulo in 2022

Institutes	Total number of teleconsultations	Medical teleconsultations	Multiprofessional teleconsultations
ICESP	58,043	27,510	30,533
ICR	4250	2444	1806
IMREA	52,765	1901	50,864
IOT	2343	2209	134
IPq	14,140	4077	10,063
InCor	3165	1383	1782
ICHC	5965^*^	—	—
Total	14,0671	45,489	95,182

*Source:* data extracted from the hospital intelligence platform (PIH). (^*^) The number of multidisciplinary teleconsultations at ICHC was counted together with medical teleconsultations. ICESP, Cancer Institute; ICR, Children Institute; IMREA, Physical Medicine and Rehabilitation Institute; IOT, Orthopedic Institute; IPQ, Psychiatry Institute; INCOR, Heart Institute; ICHC, Central Institute.

**Table 3.** Specialties That Performed Teleconsultation by Institute in 2022

Institutes	Medical specialties		Multi-professional specialties
ICESP	1. Gastrointestinal surgery 2. Thoracic surgery 3. Dermatology 4. Endocrinology 5. Hepatic gastrology 6. Vertebral column group 7. Hematology 8. Hysteroscopy 9. Infectology 10. Mastology 11. Neurology 12. Nutrilogy 13. Orthopedics 14. Otorhinolaryngology 15. Radiotherapy 16. Plastic surgery 17. Sarcoma, melanoma surgery	18. Psychiatry 19. Neurosurgery 20. Pain group 21. Palliative care 22. Physiatry/rehabilitation 23. Catheter group 24. Nephrology 25. Clinical oncology 26. Pneumology 27. Interventional medicine 28. Radiology 29. Cardiology 30. Geriatrics 31. Cosmetic gynecology 32. Head and neck surgery	1. Physical education 2. Nursing 3. Physiotherapy 4. Speech therapy 5. Nutrition 6. Psychology 7. Occupational therapy 8. Dentistry 9. Social service
ICR	1. Immunology 2. Infectology 3. Nephrology 4. Neuropediatrics 5. Pneumology 6. Rheumatology 7. RN maternity 8. Bone marrow transplant 9. Hebiatrics	10. Psychiatry 11. Chronic ambulatory 12. Children's surgery 13. Endocrinology 14. Gastroenterology 15. Medical genetics 16. Hematology 17. Hepatology	1. Nutrition and dietetics 2. Psychology 3. Nursing 4. Physiotherapy 5. Social service
IOT	1. Pediatric syndromes group 2. Paralysis group 3. Shoulder group 4. Hand group 5. Reconstruction group 6. Trauma group	7. Hip group 8. Knee group 9. Foot group 10. Osteometabolic group 11. Physiatrics group 12. Column group	1. Psychology 2. Nursing 3. Social service
IPq	1. Psychiatry 2. Medical residency group 3. Pediatrics 4. Geriatrics 5. Medical clinic		1. Social service 2. Nursing 3. Physiotherapy 4. Speech therapy 5. Nutrition 6. Psychology 7. Clinical psychologist 8. Occupational therapy
InCor	1. Lung transplant 2. Obstruction and smoking 3. Palliative care 4. Trachea 5. Anesthesiology 6. Thoracic surgery	7. Cardiovascular Surgery 8. Cardio-oncology 9. Lipids 10. Hypertension 11. Valvular heart disease 12. Atherosclerosis	1. Psychology 2. Nursing 3. Social service 4. Physiotherapy 5. Pharmacy 6. Nutrition and dietetics 7. Dentistry
ICHC	1. Endocrinology and metabology 2. Geriatrics clinical 3. Neurology 4. Pneumology 5. Anesthesiology 6. Phoniatry 7. Gynecology 8. Immunology 9. Gastrosurgery (nutrology)	10. Emergency surgery 11. Palliative care 12. Obstetrics 13. General surgery 14. Head and neck surgery 15. Dermatology 16. Infectious diseases 17. General clinic	7. Psychology 8. Physiotherapy 9. Clinical pharmacy 10. Nutrition and dietetics 11. Speech therapy

Institutes	Medical specialties	Mixed specialties	Multi-professional specialties
IMREA	1. Physitatry 2. Psychiatry 3. Urology	1. Therapeutic workshop technician 2. Musculoskeletal disorders 3. Amputees group 4. Hemophilia in children 5. Brain injury 6. Spinal cord injury 7. Neurodegenerative group 8. Neuromuscular 9. Down's syndrome	1. Physical conditioning 2. Nursing 3. Physiotherapy 4. Speech therapy 5. Nutrition 6. Dentistry 7. Psychology 8. Social servisse 9. Occupational therapy

ICESP, Cancer Institute; ICR, Children Institute; IMREA, Physical Medicine and Rehabilitation Institute; IOT, Orthopedic Institute; IPQ, Psychiatry Institute; INCOR, Heart Institute; ICHC, Central Institute.

The Net Promoter Score (NPS) was used to evaluate the patient's satisfaction with the HCFMUSP teleconsultation service. To briefly introduce the concept, the NPS is a measure of customer loyalty developed by Reichheld in 2003. This metric assumes that customers are either promoters (80–100), defined as satisfied users or detractors (0–60), unsatisfied users. These metrics can be used as indicators for qualifying service according to customers' feedback.^17^

According to our metrics, a 58 NPS was obtained in the end of 2022, indicating a good acceptance with the patients' HCFMUSP assisted. In detail, 69% gave positive feedback in regards to the easiness and quality offered by the virtual platform, 69% also approved the option to have online prescriptions, 71% answered that their expectations were matched with the teleconsultation service, and 74% approved the work done by the digital health TI team.

We estimated that more than $500,000 were saved by the patients in travel costs, considering round trips via public transport. This value could be even higher if indirect costs were considered, such as absence of working days, food, supplies, and security. We achieved this estimated amount by multiplying the number of teleconsultations performed in 2022 by the minimum value spent by the patients in round trips to health centers for face-to-face consultations, and we then divided this number by the dollar quotation on that time.

## Challenges and Solutions During Teleconsultation Implementation at the HCFMUSP

Overall, the process to implement the teleconsultation service at the HCFMUSP faced several challenges. Among them, we highlight the traditional institutional culture and the reluctance in adopting the new telehealth practices, slowing down the implementation process.

We found that educational workshops held at the HCFMUSP were essential for convincing institutional leaders by demonstrating a governance strategy focused on Digital Health and Telemedicine approaches, which helped quicken the implementation process and shape a new institutional culture. In addition to these joint actions, guided weekly virtual meetings among the institutes facilitated their integration during the teleconsultation implementation process.

Other relevant actions taken involved the investment and the development of a devoted infrastructure for the teleconsultation practices. For example, the feasibility study to create adapted spaces for different medical specialties to assist patients during the teleconsultation and facilitate the institutional adherence. The data integration among the institutes represented another important challenge to standardize the process; however, we overcame this by the implementation of the digital certification process across all the institutes.

One of the current challenges we are facing is to attract funds to expand the teleconsultation process in some of the HCFMUSP institutes and increase the services provided to other health care institutions. Therefore, we are tackling this challenge by developing and testing strategies to attract investment from partners, either from the private sector or from public funds available for the national health care system.

## Conclusions and Perspectives

Besides the promising results we have had so far, the standardization and implementation of the teleconsultations in the HCFMUSP is still an ongoing process. We noticed the need to increase the workforce and its qualification to continuously improve and expand the provided telehealth practices for the public health system, and then help to create a broad digital health culture among health care professionals and patients.

In addition, from now on we intend to introduce new technological solutions, such as telemonitoring strategies, to improve the patient's follow-up. We expect this new initiative will favor the teleconsultation by increasing the services provided and reducing even more the necessity of face-to-face consultations.

Our experience demonstrated good acceptance and satisfaction of patients with teleconsultations services. The availability of a support digital health team was essential to make teleconsultations feasible, as well as to provide individualized attention to the patient's technological needs. In addition, the digital certification and data integration in digital informative dashboards were decisive steps to conclude on the teleconsultation implementation cycle, contributing to decision making and better planning and improvements in the implementation processes.

The perspective of the HCFMUSP is to completely overhaul the patients' journey by implementing digital health practices supported by multi-professional health care, and then create a complete digital health care line and eliminate unnecessary face-to-face consulting and long and costly patient hospital trips.

We believe that a mature teleconsultation service provided by the HCFMUSP will lead to an increase of population covered by specialized health assistance, including economically deprived regions of this vast country, and contribute to an even fairer Brazilian health system. Finally, we expect that HCFMUSP experience, shared in this work and future reports, can help countries in the development to consider implementing telehealth solutions as a public health strategy.

## Abbreviations Used

AI: artificial intelligence
BHP-UK: Global Better Health Programme
COVID-19: coronavirus disease
HCFMUSP: Hospital das Clínicas da Faculdade de Medicina da Universidade de São Paulo
ICESP: Cancer Institute
ICHC: Central Institute
ICR: Children Institute
ICU: intensive care unity
IMREA: Physical Medicine and Rehabilitation Institute
InCor: Heart Institute
IOT: Orthopedic Institute
IPq: Psychiatry Institute
NPS: Net Promoter Score
SUS: Sistema Único de Saúde
